# Cucurbitacins B, E and I Concentrations and Relationship with Drought Tolerance in Bottle Gourd [*Lagenaria siceraria* (Molina) Standl.]

**DOI:** 10.3390/plants12193492

**Published:** 2023-10-07

**Authors:** Phumzile Mkhize, Hussein Shimelis, Jacob Mashilo

**Affiliations:** 1 African Centre for Crop Improvement (ACCI), University of KwaZulu-Natal, Private Bag X01, Scottsville, Pietermaritzburg 3209, South Africa; shimelish@ukzn.ac.za (H.S.); jacobmashilo@yahoo.com (J.M.); 2 Limpopo Department of Agriculture and Rural Development, Towoomba Research Centre, Agriculture Regulatory and Technology Development, Crop Science Directorate, Private Bag X1615, Bela-Bela 0480, South Africa

**Keywords:** antioxidants, cucurbitacin, drought stress, high-performance liquid chromatography

## Abstract

Bottle gourd [*Lagenaria siceraria* (Molina) Standl.]) is a relatively drought-tolerant cucurbit due to the high composition of unique biochemical compositions, including cucurbitacin. The objective of this study was to determine the concentrations of cucurbitacins in bottle gourd and their relationship to drought tolerance. The study assessed 12 bottle gourd accessions grown under two moisture levels (i.e., non-stressed (NS) and drought-stressed (DS)) and three drought stress intensities (i.e., mild, moderate, and severe) using a 12 × 2 × 3 factorial experiment designed in a randomized complete block design with three replications. Control studies were undertaken under glasshouse conditions. The content of cucurbitacins B, E, and I were quantified in leaves and roots using high-performance liquid Cchromatography–mass spectrometry (HPLC-MS). The free radical scavenging activities of pure cucurbitacins B, E, and I were quantified using 2,2-diphenyl-1-picrylhydrazyl (DPPH) and a ferrulic acid power assay (FRAP). Results revealed that cucurbitacins B and I were present in accessions BG-48, BG-58, BG-70, BG-78, BG-79, BG-81, BG-52, and GC in leaves and roots under DS condition. The contents of cucurbitacins B and I were enhanced under increased drought intensity for accessions BG-48, BG-81, and GC. In all the leaf and root samples, cucurbitacin E was not detectable. Based on the DPPH test, pure cucurbitacins I, B, and E reduced free radicals at maximum values of 78, 60, and 66%, respectively. Based on the FRAP assay, pure cucurbitacins I, B, and E had maximum ferric-reducing powers of 67, 62, and 48%. Additionally, cucurbitacin I recorded the highest antioxidant activity compared to cucurbitacins B and E. Increased cucurbitacin accumulation and antioxidant properties indicate their role in minimising cell damage caused by oxidative stress under drought-stressed environments. The present study revealed that cucurbitacins B and I serve as novel biochemical markers for screening drought tolerance in bottle gourd or related cucurbits.

## 1. Introduction

Bottle gourd [*Lagenaria siceraria* (Molina) Standl.] is an important cucurbit crop in Sub-Saharan Africa (SSA) and is widely cultivated by small-holder growers for its nutritious fruit. Young and succulent leaves are consumed as leafy vegetables after cooking and are often used for medicinal purposes to treat headaches [1,2]. Bottle gourd fruits are harvested while tender and boiled until soft for consumption. They are a source of essential nutrients, including iron, zinc, nitrogen, manganese, copper, phosphorus, potassium, calcium, and magnesium [3,4,5,6]. Sugar and milk are often added to the boiled fruit to enhance taste [7]. The fruit are a good source of vitamins—including B, C, and E—carbohydrates, crude proteins, crude lipids, phenols and dietary fiber [5,8,9,10]. The fruits are also an excellent source of essential amino acids, including glutamic acid, leucine acid, arginine, lysine and aspartic acid, threonine, serine, alanine, valine, phenylalanine and arginine [3,11,12,13]. Ripening seeds are often consumed alongside the tender fruits. The seeds are dried and roasted to be eaten as snacks or make flour [7]. The seeds contain essential amino acids, minerals—including copper, phosphorus, zinc, iron, and magnesium—and proteins [12,13]. The seed oil contains sterols, including β-sitosterols [14]. The mature fruits are often dried and used to make traditional containers called “Kgapa” or “Sego” in the indigenous and local Sepedi language of South Africa. The containers are used to store food, grains, and water or for decorative purposes [15]. Bottle gourd serves as a rootstock for grafted watermelon and improves fruit yield, quality, and resistance to *Fusarium* and *Verticilium* wilts [15,16,17,18,19].

Bottle gourd shows considerable drought tolerance when compared to other cucurbits, including pumpkin (*Cucurbita maxima*) and cucumber (*Cucurbita pepo*) [20]. In SSA, bottle gourd production occurs under extreme climatic conditions characterized by drought and heat stress. The prevailing of the crop under harsh growing conditions made it a hardy and drought-tolerant crop [20,21,22]. The ability of bottle gourd to tolerate drought stress is associated with the high composition of unique biochemical compositions, including cucurbitacin and physiological attributes [22]. It shows diverse phenotypic attributes associated with its wide adaptation and cultivation in various agro-ecological zones globally, including in Africa, India, Turkey, the USA, and China [23,24,25,26].

Cucurbit crops, including bottle gourd, produce secondary metabolites, including cucurbitacins [3,24,25,26,27]. Cucurbitacins induce a bitter taste in the leaves, roots, and fruits of most cucurbit crops and are toxic when consumed in large quantities [28,29,30,31,32,33]. The following cucurbitacins are reportedly produced in cucurbits: A, B, C, D, E, F, H, I, L, Q, R, 23, 24-dihydrocucurbitacin F, dihydrocucurbitacin B, and hexanorcucurbitacin F [34,35,36,37]. The biosynthesis of cucurbitacin varies between the *Cucurbitaceae* crops and genotypes of the same species. For example, cucurbitacin B is found in melon [38,39], C in cucumber [40], E and I in bottle gourd [41], E in watermelon [33], and IIa in *Hemsleya macrosperma* [38]. Cucurbitacins possess various pharmacological and pharmaceutical values, including neuroprotective [42], anti-inflammatory [43], anti-tumor [44], hepatoprotective [45], hypoglycemic [46], and anti-microbial functions [47], thus making bottle gourd consumption greatly beneficial for medicinal purposes. For example, an anti-cancer activity of cucurbitacins has been detected against cells associated with breast and ovarian cancer, hepatocellular carcinoma, human T cell leukemia, pancreatic cancer, colon cancer, and liver carcinoma [48]. Other pharmacological values of bottle gourd associated with the presence of cucurbitacins include managing ulcers, diabetes, diarrhea, and obesity [49].

Cucurbitacins are produced in cucurbits as a defense strategy against insect pests and diseases [50]. They are also produced in response to abiotic stress, including UV radiation, possibly as antioxidant molecules to reduce cell damage caused by reactive oxygen species (ROS) (i.e., free radicals such as O^2^, OH¯, H_2_O_2,_ and ^1^O_2_) [51,52]. In addition, cucurbitacin accumulation has been associated with increased drought tolerance [41,47,53]. Reportedly, cucumbers subjected to drought stress contained twice the amount of cucurbitacins compared to non-stressed plants [54]. Mashilo et al. [41] detected cucurbitacins E and I in bottle gourd in response to water stress. The authors reported that the enhanced production of cucurbitacins E and I under water-stressed conditions was linked to drought stress tolerance. However, there is limited information on cucurbitacins’ magnitude and expression in regulating drought and other abiotic stress in cucurbits. Therefore, understanding the magnitude and association of cucurbitacins in drought adaptation may aid novel selection markers for drought tolerance breeding in bottle gourd or related cucurbits. The objective of this study was to determine the concentrations of cucurbitacins in bottle gourd and their relationship with drought tolerance.

## 2. Results

### 2.1. Soil Moisture Content

**Figure 1:**
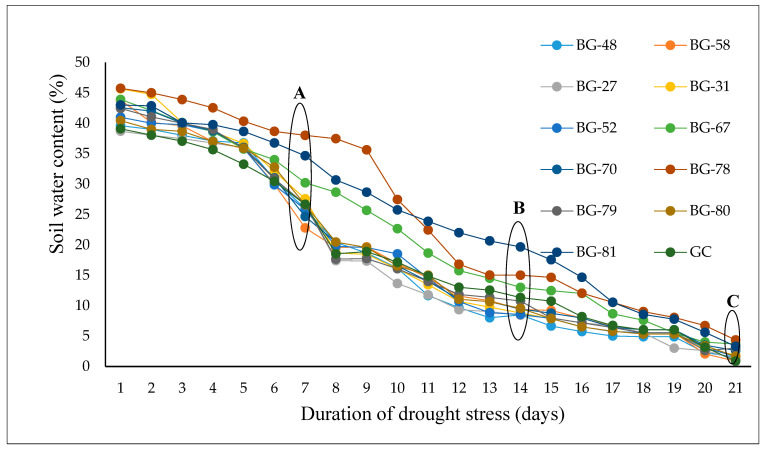
Mean soil water content (%) of the accessions grown under drought stress conditions. The areas marked A, B, and C correspond to mild, moderate, and severe drought stress intensity, respectively.

Significant differences were observed in soil water content across the twelve accessions grown under DS conditions as presented in Figure 1. The NS treatment is not shown in Figure 1, as soil water content was maintained at field capacity (~40% *v*/*v*) throughout the experiment. Under DS conditions, a steady decrease in the soil water moisture was recorded across the different accessions. At 7 days, the soil moisture level had reached approximately 25 to 30% for most accessions. At 14 days of withholding water, the soil moisture content decreased to approximately 7 to 14% for most accessions, except for BG-81, which was at approximately 21%. At 21 days, which corresponded to the severe drought stress, the soil moisture content reached approximately 0 to 5% across the different accessions.

### 2.2. Cucurbitacins B, E, and I Accumulation in Bottle Gourd Accessions under DS and NS Conditions

**Figure 2:**
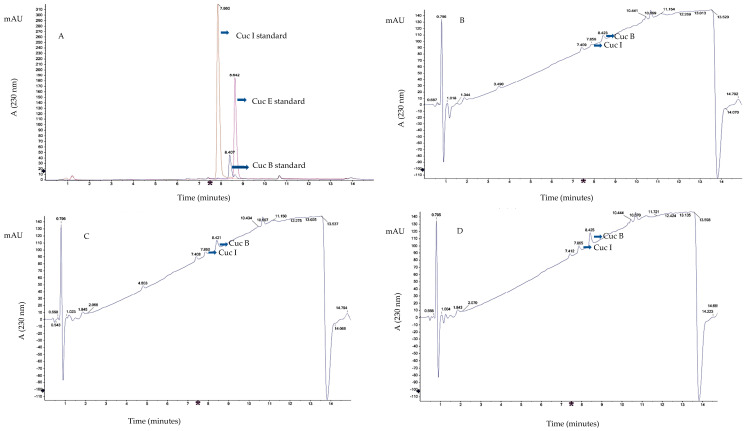
Chromatograms showing mass spectra and retention times of cucurbitacin B (Cuc B − blue), cucurbitacin E (Cuc E – purple), and cucurbitacin I (Cuc I − pink) standards (**A**) and Cuc B, Cuc E, and Cuc I detection in leaf (**B**) and root samples (**C**,**D**). mAU = milli −absorbance unit.

The contents of cucurbitacins B and I in bottle gourd leaves and roots grown under DS and NS conditions are shown in Table 1. Representative chromatograms for cucurbitacins B and I detected in samples are presented in Figure 2. Cucurbitacin E was not detected in any of the samples. Under DS condition, cucurbitacin B was detected in accessions BG-48, BG-81, BG-70, BG-78, BG-79, and GC in both root and leaf samples. Accessions BG-48, BG-81, and GC accumulated cucurbitacin B of 0.03 mg/g in the leaves under DS conditions. There were significant changes in the root cucurbitacin B concentration under increasing drought intensity for accessions BG-81, BG-48, and GC. Cucurbitacin B contents in roots for BG-81 were 0.04, 0.05 and 0.08 mg/g under mild, moderate and severe drought stress intensities, respectively. Cucurbitacin B values in the roots of BG-48 were 0.3, 0.5, and 0.6 mg/g under mild, moderate, and severe drought stress intensity, respectively. Cucurbitacin B in roots of GC were 0.03 under mild drought stress and increased to 0.04 and 0.06 mg/g under moderate and severe drought stress intensities, respectively. Under NS conditions, cucurbitacin B was not detected in the leaf samples for all accessions, whereas it was detected in the roots of accession BG-31.

Cucurbitacin I was not detected in the leaves of any of the accessions under mild drought stress. However, under moderate and severe drought stress cucurbitacin I was detected for the accessions BG-48, BG-81, and GC. In the leaf samples of the accessions BG-48, BG-81, and GC, drought stress did not induce significant changes in the accumulation of cucurbitacin I. Cucurbitacin I was not detected in leaf samples under NS conditions. Root cucurbitacin I content increased with increasing drought stress intensity for accessions BG-48 and GC, whereas no significant changes were observed for accessions BG-70, BG-79, and BG-81. Under the NS condition, cucurbitacin I was not detected in the root samples.

### 2.3. Free Radical Scavenging Activity of Pure Cucurbitacins B, E, and I

**Figure 3:**
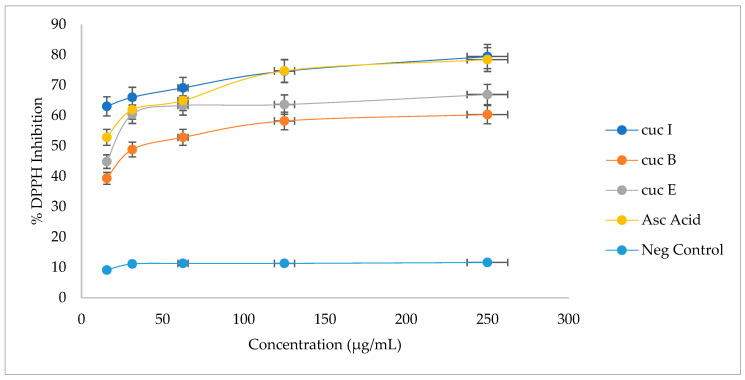
Free radical inhibiting activity of pure cucurbitacins B, E, and I based on the DPPH assay. Values are means ± standard error.

Antioxidant potential of cucurbitacins B, E, and I based on the DDPH assay is presented in Figure 3. Cucurbitacin B recorded the lowest DPPH-reducing activityies of 58 and 60% at 125 and 250 µg/mL, respectively, compared to other cucurbitacins. However, this activity was relatively higher when compared to that of the negative control, which was at a steady 12% across the different concentrations. Cucurbitacin E recorded DPPH-reducing activities of 64 and 67% at 125 and 250 µg/mL, respectively. Cucurbitacin I recorded the highest DPPH-reducing activities of 75 and 79% at 125 and 250 µg/mL, respectively, compared to the other cucurbitacins.

### 2.4. Ferric-Reducing Power of Cucurbitacins B, E, and I

**Figure 4:**
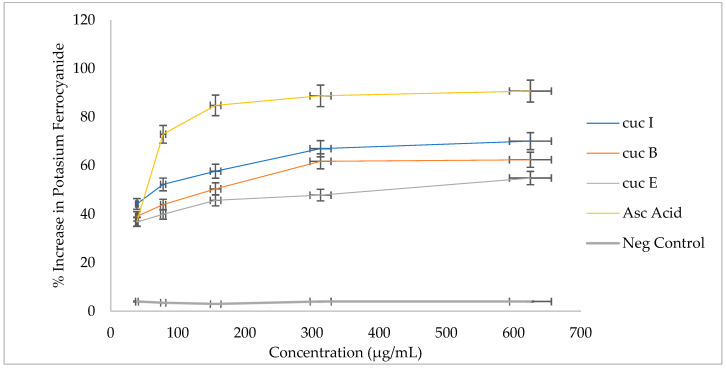
Free-radical-inhibiting activity of pure cucurbitacins B, E, and I based on their ability to convert potassium ferricyanide Fe^3+^ to Fe^2+^. Values are means ± standard error.

**Figure 5:**
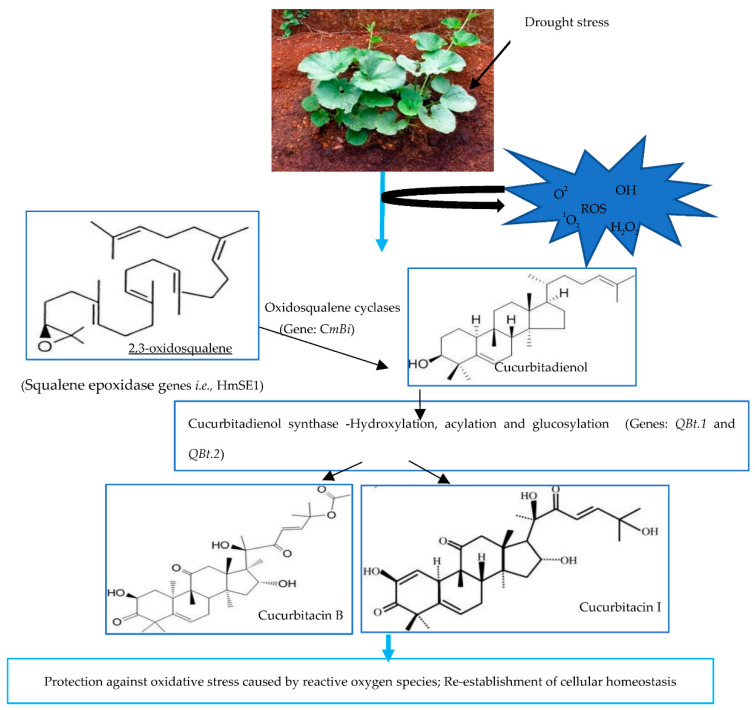
Physiological model showing critical steps involved in the synthesis of cucurbitacins B and I, chemical structures of cucurbitacins B and I, the major genes expressed, and their functions as antioxidant molecules in bottle gourd exposed to drought stress. The blue arrows represent the critical steps from drought stress exposure to protection against oxidative stress. The black arrows indicate the critical steps in cucurbitacins biosynthesis triggered by drought stress exposure, including the genes, important enzymes and molecular structures.

Antioxidative activity of cucurbitacin B, E, and I as monitored by their ability to convert potassium ferricyanide (Fe^3+^) to Fe^2+^ is presented in Figure 4. A steady increase in the ferric-reducing power was recorded across the different cucurbitacins compared to the negative control. Cucurbitacin E recorded the lowest activities of 47 and 55% at 312.5 and 625 µg/mL, respectively, compared to the other cucurbitacins. However, the activity of cucurbitacin E was relatively high when compared to that of the negative control. Cucurbitacin B recorded 62% activity at 312.5 and 625 µg/mL. Cucurbiracin I recorded the highest activity of 67% at 312.5 and 625 µg/mL. The physiological model showing the synthesis and potential involvement of cucurbitacin B and I as antioxidant compounds responsible for neutralizing ROS under drought stress conditions is shown in Figure 5.

## 3. Discussion

Cucurbitacins are a group of triterpenoids that occur exclusively in most cucurbit crops, including bottle gourd [6,30,31,32,33,44,55]. These compounds possess various human health benefits, including anti-cancer and anti-diabetic properties [1,6,7,9]. Evidence suggests drought tolerance in cucurbits could be linked to the accumulation of cucurbitacins [41]. However, there is a lack of associated data available in the literature. The current study quantified the concentrations of cucurbitacin B, E, and I in bottle gourd subjected to drought stress to elucidate their possible physiological roles in conferring drought tolerance in bottle gourd.

In the present study, cucurbitacins B and I were detected in the leaves and roots of some accessions of bottle gourd (Table 1). Increased concentrations of cucurbitacins B and I were more noticeable in the roots under increased drought intensity in some accessions, including BG-81, BG-48, and GC (Table 1). This suggested water deficit enhances the accumulation of cucurbitacins in bottle gourd. In agreement with the previous study by Mashio et al. [41], the present study reported varied contents of cucurbitacin I in bottle gourd subjected to water stress. According to Davidovich-Rikanati et al. [54] abiotic stress influences cucurbitacin biosynthesis by modulating the expression of Bi and/or Bt genes, which which catalyzes the breakdown of 2,3-oxidosqualene to cucurbitadienol to derive various cucurbitacins.

The biosynthesis of cucurbitacin B and I is conditioned by various genes (Figure 5). The exposure to drought stress probably increased cellular oxidative stress, triggering a series of biochemical reactions, including the synthesis of secondary metabolites, such as the cyclization of 2, 3-oxidosqualene by different oxidosqualene cyclases genes [56]. This is a vital step in determining the different triterpenoid backbones [56]. There are a number of genes in the oxidosqualene cyclase family such as C*m*B*i*, which catalyze the conversion of 2,3-oxidosqualene to Cucurbitadienol [34,54,57]. Cucurbitadienol synthase is the key enzyme responsible for cucurbitacin biosynthesis in cucurbit crops [58]. The activity of cucurbitadienol is controlled by the gene CYP450s [58]. In bottle gourd, the genes *QBt.1* and *QBt.2* regulate cucurbitacin biosynthesis, which is dissimilar to bitterness-regulating genes in other cucurbit crops (i.e., cucumber, melon, and watermelon) [59]. Therefore, cucurbitacin biosynthesis is dependent on the type of cucurbit species. The genes *QBt.1* and *QBt.2* could probably enhance the concentration of cucurbitacins B and I in the leaves and roots of bottle gourd [41] subjected to drought stress (Figure 5), similar to the findings of this study.

In the present study, cucurbitacin B and I did not accumulate in most of the bottle gourd accessions, suggesting accession-specific accumulation of the cucurbitacins. This is associated with a preferential selection pressure for the non-bitter types in bottle gourd for various uses, including food. For example, the tender leaves of bottle gourd are cooked as leafy vegetables by the Venda tribe of South Africa [60]. The consumption of the leaves in some parts of sub-Saharan Africa suggests possible loss of bitterness during crop domestication [61]. The roots of bottle gourd are extremely bitter, an indication of high amounts of cucurbitacins. However, in the present study, cucurbitacins B and I in roots were not detected in most of the studied accessions (Table 1), except for BG-48, BG-53, BG-48, BG-70, and GC. This indicates that cucurbitacin accumulation is accession-specific, perhaps due to varied expression of biosynthetic genes [54]. For example, the genes designated *CmBi*, *Cm710*, and *CmAC* showed varied expression to regulate cucurbitacin B contents in melon (*Cucumis melo*) [52]. In watermelon (*Citrullus lanantus* var. *lanantus*), genotypic-specific accumulation of cucurbitacins B and E has been reported [62]. Accessions BG-48, BG-81, and GC, which accumulated high amounts of cucurbitacin B and I, were previously identified as drought-tolerant by Mashilo et al. [41], perhaps due to their cucurbitacin accumulation and other physiological mechanisms, including efficient photosynthetic capacity and photo-protection.

To determine the possible roles of cucurbitacins as antioxidant molecules, pure cucurbitacin samples were evaluated for their antioxidant activity using DPPH and FRAP assays (Figure 3 and Figure 4). DPPH radical is a stable free radical due to its unpaired electron delocalization over the whole molecule. The donation of a proton (H^+^) to this radical causes a color change from violet to pale yellow in a solution [63]. The yellow color development results from the odd electron of a nitrogen atom in DPPH being reduced by receiving a hydrogen atom from antioxidants [64,65]. The Ferric-reducing ability of a compound is monitored through the reaction between the “compounds of interest”—in our case, cucurbitacin with potassium ferricyanide (Fe^3+^)— to form Fe^2+^. The potassium ferrocyanide then reacts with ferric chloride to form a ferric-ferrous complex, a reaction monitored through the color change from yellow to green [66]. To determine the possible roles of cucurbitacins as antioxidant molecules, pure cucurbitacin samples were evaluated for their antioxidant activity using DPPH and FRAP assays (Figure 3 and Figure 4). Amongst the three pure cucurbitacin solutions, cucubitacin I recovered the highest activity based on both assays, indicating potential radical-scavenging activity and conferring drought stress tolerance by reducing cell and tissue damage associated with oxidative stress under drought stress [67,68]. Drought tolerance in maize and sorghum were associated with increased antioxidant activity which correlated with less cell damage under water-deficit conditions [68]. Increased antioxidant activity in wheat was also associated with drought tolerance [69]. In melon (*Cucumis melo* L.), cucurbitacin B accumulation has been associated with increased antioxidative activity that enhanced tolerance to biological stress [66]. Cucurbitacin-I-, B-, and E-enriched Cucurbitaceae crops, such as *Blastania cerasiformis* (Stocks) A. Meeuse and *B. garcinii* (Burm.f.) Cogn., were associated with high antioxidant activity [29]. In citron watermelon (*Citrullus lanatus* var. *citroides*), cucurbitacin E was reported to be an inefficient scavenger of reactive oxygen species [70,71,72]. In the present study, an increase in the concentrations of cucurbitacins B and I is associated with some level of drought stress tolerance in bottle gourd and serves as a potential marker for selecting drought-tolerant accessions for breeding. There is a need for genetic analysis, including the roles and expression in the biosynthesis of cucurbitacins B, E, and I under abiotic stress conditions. Additionally, the detection and possible involvement of other cucurbitacins in conferring abiotic stress tolerance in bottle gourd is paramount.

## 4. Materials and Methods

The study used 12 selected accessions of bottle gourd assigned the following codes by the Limpopo Department of Agriculture and Rural Development (LDARD) namely: BG-27, BG-31, BG-48, BG-52, BG-58, BG-67, BG-70, BG-78, BG-79, BG-80, BG-81, and GC. BG stands for botte (B) gourd (G), whereas the numbers denote the accession entry number based on the collection mission. These accessions possess varying drought tolerance levels as reported in our previous work [22,41]. Furthermore, the accessions are commonly cultivated in the Limpopo Province of South Africa by small-holder farmers under dryland, high-drought, and heat stress conditions. Entry GC is grown in KwaZulu-Natal Province and sold in retail stores in South Africa. The landrace accessions are maintained by LDARD at Towoomba Agricultural Development Centre (TADC), Bela-Bela, South Africa.

### 4.1. Experimental Design and Sample Collection

The experiment was conducted during the 2021 season under a growth chamber environment at the University of Limpopo (−25°36′54″ S, 28°0′59.76″ E, 1312 m above sea level), South Africa. The study used the 12 bottle gourd accessions grown under two moisture levels (i.e., non-stressed (NS) and drought-stressed (DS)) and three drought stress intensities (i.e., mild, moderate, and severe) using a randomized complete block design with 3 replications. Seeds of the accessions were grown in a 2 L capacity polyethylene plastic pot containing approximately 2 kg of loamy soil collected from the University of Limpopo, Syferskuil Experimental farm (−23°53′9.60″ S, 29°44′16.80″ E, 1312 m above sea level). Three seeds were sown per accession and were watered daily to maintain soil moisture content at approximately 40% *v*/*v* field capacity. One plant was maintained per pot upon germination and three weeks after emergence. The plants were watered with tap water until a sixth fully expanded leaf was developed approximately 18 to 22 days after planting. Water was then withheld for DS treatment for the entire duration of the experiment, whereas the NS plants were supplied with water. The experimental units were without water on days 7, 14, and 21, corresponding to mild, moderate, and severe drought stress, in that order. Three fully expanded leaves were collected from each plant in DS and NS conditions. The same plants used for leaf sampling were carefully exhumed to expose the roots. The roots were gently washed with tap water to remove soil debris. The leaf and root samples were placed in 50 mL centrifuging tubes and kept at −80 °C in a −86 °C ultralow freezer until analysis.

### 4.2. Sample Extraction and Determination of Cucurbitacins

Cucurbitacin extraction was carried out as previously outlined [41,73] with some modifications. Extraction of cucurbitacins from the leaf and root samples was carried out by grinding 0.1 g of the samples to powder using a pestle and mortar in liquid nitrogen. The ground sample was transferred to a 2 mL Eppendorf tube. To each sample, 1 mL of ice-cold methanol (HPLC-grade, Sigma Aldrich) was added, and the mixture homogenised for 15 min using a mini-bead-beater (Biospec Bartlesville, OK, USA). After that, the mix was centrifuged at 14,000× *g*, 15 min at 4 °C. The supernatant was removed and filtered through hydrophilic polypropylene membrane (13 mm × 0.2 μm) and the cucurbitacin-containing extract was stored at −20 °C in a freezer.

### 4.3. Detection of Cucurbitacins

**Figure 6:**
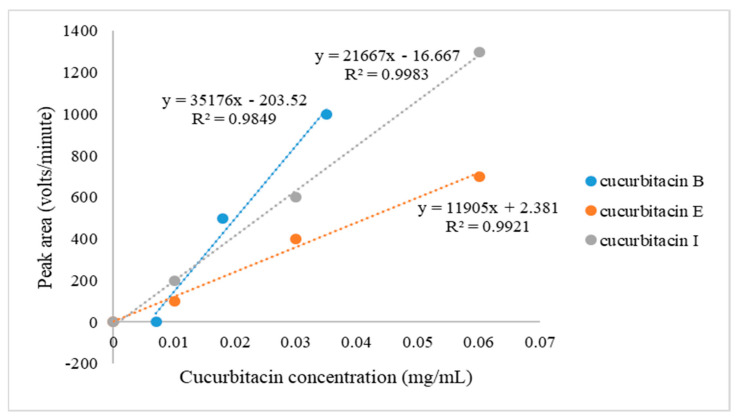
Calibration curve showing the peak areas of the standard cucurbitacins B, E, and I of a known concentration range of 0−0.1 mg/mL, used to quantify the unknown concentration of each of the cucurbitacins (B, E, and I) in the fully expanded leaf and whole root samples of 12 bottle gourds grown under drought-stressed and non-stressed conditions.

The cucurbitacin analyses were performed at the Department of Chemistry, School of Physical and Mineral Sciences, University of Limpopo, South Africa. Cucurbitacin analyses were performed using an SCIEX ExionLC-UV detector, LC, equipped with a Phenomenex Luna C18 2.5 µm, 2 × 100 mm column. The mobile phase consisted of 0.1% formic acid in water (A) and 0.1% of formic acid in methanol (B). Gradient elution was as follows: 0–0.5 min 20% B, 0.5–10 min 90% B, 10–12.50 min 90% B, 12.50–12.60 min 20%, 12.60–15 min 20% B (run time 15 min). Flow rate was maintained at 0.4 mL/min at 40 °C. Cucurbitacin identification was performed via the comparison of the retention times of the cucurbitacin B, E, and I standards detected at 230 nm. Pure cucurbitacin B, E, and I standards were purchased from Sigma-Aldrich (South Africa, South Modderfontein, Johannesburg). The samples were extracted from *Luffa operculate* (L.) Cohn, and isolation was conducted using a column chromatography. Molecular identification was performed with nuclear magnetic resonance. Cucurbitacins B, E, and I were quantified using an external calibration curve on a dry weight basis (Figure 6). Figure 6 shows the calibration curve constructed from peak areas of the three reference standards, i.e., cucurbitacins B, E, and I versus their concentrations, used to quantify the unknown concentrations of the cucurbitacins in leaves and roots of 12 bottle gourd genotypes grown under drought stress and non-stress conditions.

### 4.4. Quantification of Antioxidant Levels of Pure Cucurbitacins B, E, and I

#### 4.4.1. 2,2−Diphenyl−1−Picrylhydrazyl (DPPH) Assay

The free radical scavenging activities of pure cucurbitacins B, E, and I were determined using the 2,2-diphenyl-1-picrylhydrazyl (DPPH) assay as previously outlined [74] with some modifications. Cucurbitacin standards were dissolved in methanol (HLPC grade—Sigma Aldrich) to prepare an initial 250 µg/mL solution. From the initial solution, different solutions of concentrations ranging from 250 µg/mL to 15.63 µg/mL to a final volume of 1 mL were prepared in triplicates. The concentration range consisted of the following: 250.00, 125.00, 62.50, 31.25, and 15.63 µg/mL. L-ascorbic acid was used as a standard (positive control) by preparing a range of the same concentration as the cucurbitacin standards. To the 1 mL solutions of cucurbitacins and the positive control, 2 mL of 0.2 mmol/L DPPH solution (dissolved in methanol) was added and vortexed thoroughly. The negative control solution was prepared by adding 2 mL of 0.2 mmol/L DPPH to 1 mL of distilled water. All the mixtures were kept at room temperature in the dark for 30 min. After the elapsed time, the absorbance of each solution was measured at 517 nm using DU^®^ 730 Life Science UV/Vis spectrophotometer (Beckman Coulter, Canada, Mississauga, ON, Canada). The absorbance reading was averaged for each of the samples, and the percentage antioxidant potential (percentage inhibition) was calculated using the following formula [75]:

(1) $$\%\;Inhibition=\left[\frac{Ac-As}{Ac}\right]\times 100$$

where

*Ac* = Absorbance of control (negative) solution
*As* = Absorbance of cucurbitacin solution

#### 4.4.2. Ferric-Reducing Power Assay

Evaluation of the ferric-reducing power of cucurbitacins B, E, and I was carried out as previously outlined [76] with some modifications. Different concentrations of each cucurbitacin standard were prepared in triplicate to the final concentrations of 625.00, 312.50, 156.25, 78.13, and 39.06 μg/mL to a total volume of 2.5 mL. The same concentrations of L-Ascorbic acid were prepared, serving as a positive control. The different concentrations were mixed with 2.5 mL of sodium phosphate buffer (0.2 M, pH 6.6) and 2.5 mL of potassium ferricyanide (Rochelle, Johannesburg, South Africa) (1% *w*/*v*) in distilled water in test tubes. After adding each solution, the mixtures were vortexed for approximately 10 s. This was followed by incubation at 50 °C for 20 min. After that, 2 mL of trichloroacetic acid (Sigma, Johannesburg, South Africa) (10% *w*/*v*) in distilled water was added to each test tube. The tubes were left to stand at room temperature for approximately 20 min; during this time, the sediments settled at the bottom of the tube and the yellow top parts of the mixtures were transferred to new, clean test tubes. To the yellow solution in a new test tube, 5 mL of distilled water and 1 mL ferric chloride (0, 1% *w*/*v*) in distilled water were added, and the mixture was vortexed. The same procedure was followed for the preparation of the negative control; instead of adding cucurbitacin, distilled water was added. The color change for all the mixtures from yellow to green or blue was monitored and measured at a wavelength of 700 nm using DU^®^ 730 Life Science UV/Vis spectrophotometer. The percentage increase of the potassium ferrocyanide (Fe^2+^), which is directly proportional to the antioxidative power of the compound, was calculated using the following formula [77]:

(2) $$\%\;increase\;in\;potassium\;ferrocyanide=\left[\frac{As-Ac}{As}\right]\times 100$$

where

*Ac* = Absorbance of control (negative) solution
*As* = Absorbance of cucurbitacin solution

## 5. Limitations of the Study

The plant population of bottle gourd was relatively low due to larger within- and between-row planting space, limiting genotype evaluation under variable drought tolerance and multiple sampling. The collection of variable age leaf and root samples (e.g., young, matured, and relatively old) could affect the accumulation patterns and estimation of cucurbitacin contents. Furthermore, data presented in one season could limit the repeatability of the experiment and an overestimation of measurements needing multiple sampling across environments.

## 6. Conclusions

The present study determined the potential roles of cucurbitacins in conferring drought tolerance in bottle gourd. Accumulation of cucurbitacins B and I were identified in leaves and roots of some bottle gourd accessions subjected to drought stress. Based on the DDPH test and FRAP assays, pure cucurbitacins B, E, and I reduced free radicals, indicating their ability as antioxidant molecules in reducing cell damage caused by reactive oxygen species in drought-exposed bottle gourd crops. The present study revealed that cucurbitacins B and I are novel biochemical markers for screening drought tolerance in bottle gourd or related cucurbits.

## Figures and Tables

**Table 1:** Cucurbitacin B and I detections and concentrations (mg/g) in leaves and roots of bottle gourd accessions evaluated under variable drought stress intensities and non-stressed conditions.

Cucurbitacin B (mg/g)								
		Leaves			Roots		Leaves	Roots
Accession	Mild	Moderate	Severe	Mild	Moderate	Severe	NS	NS
BG-27	ND	ND	ND	ND	ND	ND	ND	ND
BG-31	ND	ND	ND	ND	ND	ND	ND	0.03
BG-48	ND	0.03	0.03	0.03	0.05	0.06	ND	ND
BG-52	ND	ND	ND	ND	ND	ND	ND	ND
BG-58	ND	ND	ND	0.03	0.03	0.03	ND	ND
BG-67	ND	ND	ND	ND	ND	ND	ND	ND
BG-70	ND	ND	ND	0.03	0.03	0.03	ND	ND
BG-78	ND	ND	ND	ND	0.03	ND	ND	ND
BG-79	ND	ND	ND	0.03	0.03	0.03	ND	ND
BG-80	ND	ND	ND	ND	ND	ND	ND	ND
BG-81	ND	0.03	0.03	0.04	0.05	0.08	ND	ND
GC	ND	0.03	0.03	0.03	0.04	0.06	ND	ND
**Cucurbitacin I (mg/g)**								
		**Leaves**			**Roots**		**Leaves**	**Roots**
**Accession**	**Mild**	**Moderate**	**Severe**	**Mild**	**Moderate**	**Severe**	**NS**	**NS**
BG-27	ND	ND	ND	ND	ND	ND	ND	ND
BG-31	ND	ND	ND	ND	ND	ND	ND	ND
BG-48	ND	0.04	0.04	0.03	0.04	0.5	ND	ND
BG-52	ND	ND	ND	0.03	0.03	0.03	ND	ND
BG-58	ND	ND	ND	0.03	ND	ND	ND	ND
BG-67	ND	ND	ND	ND	ND	ND	ND	ND
BG-70	ND	ND	ND	0.03	0.03	0.03	ND	ND
BG-78	ND	ND	ND	ND	ND	ND	ND	ND
BG-79	ND	ND	ND	0.03	0.03	0.03	ND	ND
BG-80	ND	ND	ND	ND	ND	ND	ND	ND
BG-81	ND	0.03	0.03	ND	0.04	0.04	ND	ND
GC	ND	0.03	0.04	0.04	0.05	0.07	ND	ND

ND = not detected, DS = drought stress condition, NS = non-stressed condition.

## Data Availability

All data is presented on the paper.

## References

[1] Moustafa S., Gabr N.M., Zaki J.K., Awdan E., Mina S.A. (2021). The anti-inflammatory, anti-ulcer activities and phytochemical investigation of *Cucumis melo* L. cv. Ismailawi fruits. Nat. Prod. Res..

[2] Behera K., Mandal U., Panda M., Mohapatra M., Mallick S.K., Routray S. (2021). Ethnobotany and folk medicines used by the local healers of Bhadrak, Odisha, India. Egypt. J. Bot..

[3] Attar U.A., Ghane S.G. (2018). Optimized extraction of anti-cancer compound—Cucurbitacin I and LC–MS identification of major metabolites from wild Bottle gourd (*Lagenaria siceraria* (Molina) Standl.). S. Afr. J. Bot..

[4] Attar U.A., Ghane S.G. (2019). In vitro antioxidant, antidiabetic, antiacetylcholine esterase, anticancer activities and RP-HPLC analysis of phenolics from the wild bottle gourd (*Lagenaria siceraria* (Molina) Standl.). S. Afri. J. Bot..

[5] Mahapatra S., Sureja A.K., Behera T.K., Bhardwaj R., Verma M. (2023). Variability in antioxidant capacity and some mineral nutrients among ninety-one Indian accessions of bottle gourd [*Lagenaria siceraria* (Molina) Standl.]. S. Afri. J. Bot..

[6] Saurabh C.K., Ghosh S.K., Sanyal B. (2023). Novel detection method to rapidly quantify toxic cucurbitacin in *Lagenaria siceraria* (bottle gourd). J. Food Sci. Technol..

[7] Barot A., Pinto S., Balakrishnan S., Prajapati J.P. (2015). Composition, Functional Properties and Application of Bottle Gourd in Food Products. Res. Rev. J. Dairy Sci. Technol..

[8] Ojiako O.A., Igwe C.U. (2007). Nutritional and anti-nutritional compositions of *Cleome rutidosperma*, *Lagenaria siceraria*, and *Cucurbita maxima* seeds from Nigeria. J. Med. Food..

[9] Upaganlawar A., Ramchandran R. (2009). Bottle gourd (*Lagenaria Siceraria*) “A vegetable food for human health”—A comprehensive review. J. Pharmacol..

[10] Sithole J.N., Modi A.T., Pillay K. (2015). An assessment of minerals and protein contents in selected South African bottle gourd landraces [*Lagenaria siceraria* (Mol.) Standl.]. J. Hum. Ecol..

[11] Chung H., Choi Y., Shin Y., Youn S. (2000). Chemical composition, quality evaluation and characteristics of immature fruits of Korean native bottle gourd (*Lagenaria siceraria*). Korean J. Hortic. Sci. Technol..

[12] Ogunbusola E.M. (2018). Nutritional and anti-nutritional composition of calabash and bottle gourd seed flours (var *Lagenaria siceraria*). J. Culin. Sci. Technol..

[13] Ogunbusola M.E., Fagbemi T.N., Osundahunsim O.F. (2010). Amino acid composition of *Lagenaria siceraria* seed flour and protein fractions. J. Food Sci. Technol..

[14] Abdel-Razek A.G., Badr A.N., Alharthi S.S., Selim K.A. (2021). Efficacy of Bottle Gourd Seeds’ Extracts in Chemical Hazard Reduction Secreted as Toxigenic Fungi Metabolites. Toxins.

[15] Mashilo J., Shimelis H., Odindo A. (2015). Genetic diversity of bottle gourd (*Lagenaria siceraria* (Molina) Standl.) landraces of South Africa assessed by morphological traits and simple sequence repeat markers. S. Afr. J. Plant Soil.

[16] Yetişir H., Sari N. (2003). Effects of different rootstock on plant growth, yield and quality of watermelon. Aust. J. Exp. Agric..

[17] Çandir E., Yetişir H., Karaca F., Üstün D. (2013). Phytochemical characteristics of grafted watermelon on different bottle gourds (*Lagenaria siceraria*) collected from the Mediterranean region of Turkey. Turk. J. Agric..

[18] Keinath A.P., Hassell R.L. (2014). Control of *Fusarium* wilt of watermelon by drafting onto bottle gourd or interspecific hybrid squash despite colonization of rootstocks by *Fusarium*. Plant Dis..

[19] Morales C., Riveros-Burgos C., Espinoza Seguel F., Maldonado C., Mashilo J., Pinto C., Contreras-Soto R.I. (2023). Rootstocks Comparison in Grafted Watermelon under Water Deficit: Effects on the Fruit Quality and Yield. Plants.

[20] Sithole N., Modi A.T. (2016). Responses of selected bottle gourd [*Lagenaria siceraria* (Molina Standly)] landraces to water stress. Acta Agric. Scand. B Soil Plant Sci..

[21] Mashilo J., Shimelis H., Odindo A. (2017). Drought tolerance of selected bottle gourd [*Lagenaria siceraria* (Molina) Standl.] landraces assessed by leaf gas exchange and photosynthetic efficiency. Plant Physiol. Biochem..

[22] Mashilo J., Shimelis H., Odindo A. (2016). Yield-based selection indices for drought tolerance evaluation in selected bottle gourd [*Lagenaria siceraria* (Molina) Standl.] landraces. Acta Agric. Scand. B Soil Plant Sci..

[23] Morimoto Y., Mvere B., Grubben G.J.H., Denton O.A. (2004). Lagenaria siceraria. Vegetables Plant Resources of Tropical Africa 2.

[24] Morimoto Y., Maundu P., Fujimaki H., Morishima H. (2005). Diversity of landraces of the white-flowered gourd (*Lagenaria siceraria*) and its wild relatives in Kenya: Fruit and seed morphology. Gen. Resour. Crop Evol..

[25] Mashilo J., Shimelis H., Odindo A. (2017). Phenotypic and genotypic characterization of bottle gourd [*Lagenaria siceraria* (Molina) Standl.] and implications for breeding: A Review. Sci. Hortic..

[26] Yetişir H., Karaca F. (2018). Assessment of rooting capacity and rootstock potential of some Turkish bottle gourd (*Lagenaria siceraria*) accessions used as rootstocks for watermelon [*Citrullus lanatus* (Thunb.) Matsum. Nakai]. Asian Res. J. Agric..

[27] Patel S.B., Attar U.A., Sakate D.M., Ghane S.G. (2020). Efficient extraction of cucurbitacins from *Diplocyclos palmatus* (L.) C. Jeffrey: Optimization using response surface methodology, extraction methods and study of some important bioactivities. Sci. Rep..

[28] Patel S.B., Ghane S.G. (2021). Phyto-constituents profiling of Luffa echinata and in vitro assessment of antioxidant, anti-diabetic, anticancer and anti-acetylcholine esterase activities. Saudi J. Biol. Sci..

[29] Attar U.A., Ghane S.G., Chavan N.S., Shiragave P.D. (2022). Simultaneous detection of anticancer compounds (Cucurbitacin I, B and E) and some pharmacological properties of Indian *Blastania* species. S. Afr. J. Bot..

[30] Sharma A., Sharma J.P., Jindal R., Kaushik R.M. (2006). Bottle gourd poisoning. Kuwait J. Sci..

[31] Cynthia H., Michael G., Shin-Pin P., Helen H. (2014). Bitter bottle gourd (*Lagenaria siceraria*) toxicity. J. Emerg. Med..

[32] Verma A., Jaiswal S. (2015). Bottle gourd (*Lagenaria siceraria*) juice poisoning. World J. Emerg. Med..

[33] Gong C., Zhu H., Lu X., Yang D., Zhao S., Umer M.J. (2021). An integrated transcriptome and metabolome approach reveals the accumulation of taste-related metabolites and gene regulatory networks during watermelon fruit development. Planta..

[34] Chen J.C., Chiu M.H., Nie R.L. (2005). Cucurbitacins and cucurbitane glycosides: Structures and biological activities. Nat. Prod. Rep..

[35] Haq F., Ali A., Khan M.N. (2019). Metabolite profiling and quantitation of cucurbitacins in *Cucurbitaceae* plants by Liquid Chromatography coupled to Tandem Mass Spectrometry. Sci. Rep..

[36] Dong L., Almeida A., Pollier J., Khakimov B., Bassard J.-E., Miettinen K. (2021). An independent evolutionary origin for insect deterrent cucurbitacins in *Iberis amara*. Mol. Biol. Evol..

[37] Zhang Y., Zeng Y., An Z., Lian D., Xiao H., Zhang R.R., Zhai F., Liu H. (2022). Comparative transcriptome analysis and identification of candidate genes involved in cucurbitacin IIa biosynthesis in *Hemsleya macrosperma*. Plant Physiol. Biochem..

[38] Xu Y., Zhang H., Zhong Y., Jiang N., Zhong X., Zhang Q. (2022). Comparative genomics analysis of bHLH genes in cucurbits identifies a novel gene regulating cucurbitacin biosynthesis. Hortic. Res..

[39] Ansari W.A., Atri N., Ahmad J., Qureshi M.I., Singh B., Kumar R., Pandey S. (2019). Drought mediated physiological and molecular changes in muskmelon (*Cucumis melo* L.). PLoS ONE.

[40] Shang Y., Ma Y., Zhou H., Zhang L., Duan H., Chen J., Zeng Q., Zhou S., Wang W., Gu M. (2014). Biosynthesis, regulation, and domestication of bitterness in cucumber. Plant Sci..

[41] Mashilo J., Odindo A., Shimelis H., Musenge P., Tesfay S.Z., Magwaza L.S. (2018). Photosynthetic response of bottle gourd [*Lagenaria siceraria* (Molina) Standl.] to drought stress; Relationship between cucurbitacins accumulation and drought tolerance. Hortic. Sci..

[42] Gitler A.D., Dhillon P., Shorter J. (2017). Neurodegenerative disease: Models, mechanisms, and a new hope. Dis. Model. Mech..

[43] Peters R.R., Farias M.R., Ribeiro-do-Valle R.M. (1997). Anti-inflammatory and analgesic effects of cucurbitacins from Wilbrandia ebracteata. J. Pharmacol..

[44] Ma W., Xiang Y., Yang R., Zhang T., Xu J., Wu Y., Liu X., Xiang K., Zhao H., Liu Y. (2019). Cucurbitacin B induces inhibitory effects via the CIP2A/PP2A/C-KIT signalling axis in t (8;21) acute myeloid leukemia. J. Pharmacol. Sci..

[45] Wynn T.A. (2008). Cellular and molecular mechanisms of fibrosis. J. Phathol..

[46] Volpe C.M.O., Villar-Delfino P.H., Anjos P.M.F.D., Machado J.A.N. (2018). Cellular death, reactive oxygen species (ROS) and diabetic complications. Cell Death Dis..

[47] Dwijayanti D.R., Shimada T., Ishii T., Okuyama T., Ikeya Y., Mukai E., Nishizawa M. (2020). Bitter melon fruit extract has a hypoglycemic effect and reduces hepatic lipid accumulation in *ob/ob* mice. Phytother. Res..

[48] Wakimoto N., Yin D., O’Kelly J., Haritunians T., Karlan B., Said J., Xing H., Koeffeler H.P. (2008). Cucurbitacin B has a potent antiproliferative effect on breast cancer cells in vitro and in vivo. J. Cancer.

[49] Balkema-Boomstra A.G., Zijlstra S., Verstappen F.W.A., Inggamer H., Mercke P.E., Jongsma M.A. (2003). Role of Cucurbitacin C in resistance to spider mite (*Tetranychus urticae*) in cucumber (*Cucumis sativus L*.). J. Chem. Ecol..

[50] Tewari R.K., Kumar P., Sharma P.N. (2006). Antioxidant responses to enhanced generation of superoxide anion radical and hydrogen peroxide in the copper-stressed mulberry plants. Planta.

[51] Mazid M., Khan T.A., Mohammad F. (2011). Role of secondary metabolites in defense mechanisms of plants. Biol. Med..

[52] Luo F., Li Q., Yu L., Wang C., Qi H. (2020). High concentrations of CPPU promotes cucurbitacin B accumulation in melon (*Cucumis melo* var. makuwa Makino) fruit by inducing transcription factor CmBt. Plant Physiol. Biochem..

[53] Haynes R.L., Jones C.M. (1975). Wilting and damage to cucumber by spotted and stripes cucumber beetles. HortScience.

[54] Davidovich-Rikanati R., Shalev L., Baranes N., Meir A., Itkin M., Cohen S., Zimbler K., Portnoy V., Ebizuka Y., Shibuya M. (2015). Recombinant yeast as a functional tool for understanding bitterness and cucurbitacin biosynthesis in watermelon (*Citrullus spp*.). Yeast.

[55] Semenya S.S., Maroyi A. (2019). Ethnobotanical survey of plants used by Bapedi traditional healers to treat tuberculosis and its opportunistic infections in the Limpopo Province, South Africa. S. Afr. J. Bot..

[56] Phillips D.R., Rasbery J.M., Bartel B., Matsuda S.P.T. (2006). Biosynthetic diversity in plant triterpene cyclization. Curr. Opin. Plant Biol..

[57] Thimmappa R., Geisler K., Louveau T., O’Maille P., Osbourn A. (2014). Triterpene biosynthesis in plants. Annu. Rev. Plant Biol..

[58] Shibuya M., Adachi S., Ebizuka Y. (2004). Cucurbitadienol synthase, the first committed enzyme for cucurbitacin biosynthesis, is a distinct enzyme from cycloartenol synthase for phytosterol biosynthesis. Tetrahedron.

[59] Wu X., Wu X., Wang Y., Wang B., Lu Z., Xu P., Li G. (2019). Molecular Genetic Mapping of Two Complementary Genes Underpinning Fruit Bitterness in the Bottle Gourd (*Lagenaria siceraria* [Mol.] Standl.). Front. Plant Sci..

[60] Zhou Y., Cun Z., Hu H., Chen J. (2016). Convergence and divergence of cucurbitacin biosynthesis and regulation in *Cucurbitaceae*. Native Plants.

[61] Kim Y.C., Choi D., Zhang C., Liu H., Lee S. (2018). Profiling cucurbitacins from diverse watermelons (*Citrullus* spp.). Hortic. Environ. Biotechnol..

[62] Irshad M.D., Zafaryab M.D., Singh M.A.N., Rizvi M.M.A. (2012). Comparative analysis of the antioxidant activity of *Cassia fistula* extracts. Int. J. Med. Chem..

[63] Ionita P. (2021). The chemistry of DPPH· free radical and congeners. Int. J. Mol. Sci..

[64] Gulcin L., Alwasel S. (2023). DPPH radical scavenging assay. Processes.

[65] Vijayalakshmi M., Ruckmani K. (2016). Ferric reducing anti-oxidant power assay in plant extract. Bangladesh J. Pharmacol..

[66] Oktay M., Gulcin I., Kufrevioglu O.I. (2003). Determination of in vitro antioxidant activity of fennel (*Foeniculum vulgare*) seed extracts. LWT Food Sci. Technol..

[67] Chanda S., Dave R. (2009). In vitro models for antioxidant activity evaluation and some medicinal plants possessing antioxidant properties: An overview. Afr. J. Microbiol. Res..

[68] Elbasheir A.A.E., Ndiko L. (2021). Antioxidant responses are associated with differences in drought tolerance between maize and sorghum. J. Oasis Agric. Sustain. Dev..

[69] Wang J., Zhang X., Han Z., Feng H., Wang Y., Kang J., Han X., Wang L., Wang C., Li H. (2022). Analysis of physiological indicators associated with drought tolerance in wheat under drought stress and re-watering conditions. Antioxid. Act..

[70] Azin Z., Emamjomeh A., Esmaeilzadeh Bahabadi S., Hasanein P. (2021). Effect of exogenous chitosan, salicylic acid, and their combination on some physiological parameters of *Citrullus colocynthis* (L.) under drought stress. Iran. J. Plant Physiol..

[71] Kang L., Wu Y., Zhang J., An Q., Zhou C., Li D., Pan C. (2022). Nano-selenium enhances the antioxidant capacity, organic acids and cucurbitacin B in melon (*Cucumis melo* L.) plants. Ecotoxicol. Environ. Saf..

[72] Abdelwahab S.I., Hassan L.E.A., Sirat H.M., Yagi S.M.A., Mohan K.S., Taha M.M.E., Ahmad S., Narrima C.S.C.P., Rais M.M., Hadi A.H. (2011). Anti-inflammatory activities of cucurbitacin E isolated from Citrullus lanatus var. citroides: Role of reactive nitrogen species and cyclooxygenase enzyme inhibition. Fitoterapia.

[73] Chigayo K., Mojapelo P.E.L., Moleele S.M. (2016). Phytochemical and antioxidant properties of different solvent extracts of *Kirkia wilmsii* tubers. Asian Pac. J. Trop. Biomed..

[74] Bondet V., Brand-Williams W., Berset C. (1997). Kinetics and mechanisms of antioxidant activity using the DPPH free radical method. J. Food Sci. Technol..

[75] Ahmed A.S., Elgorashi E.E., Moodley N., McGaw L.J., Naidoo V., Eloff J.N. (2012). The antimicrobial, antioxidative, anti-inflammatory activity and cytotoxicity of different fractions off our South African *Bauhinia* species used traditionally to treat diarrhoea. J. Ethnopharmacol..

[76] Benzie I.F., Strain J.J. (1996). The ferric reducing ability of plasma (FRAP) as a measure of “antioxidant power”: The FRAP assay. Anal. Biochem..

[77] Tannanin-Spitz T., Bergman M., Grossman S. (2007). Cucurbitacin glucosides: Antioxidant and free-radical scavenging activities. Biochem. Biophys. Res. Commun..

